# Clinical and Microbiological Characterization of Invasive Pulmonary Aspergillosis Caused by *Aspergillus lentulus* in China

**DOI:** 10.3389/fmicb.2020.01672

**Published:** 2020-07-28

**Authors:** Shu-Ying Yu, Li-Na Guo, Meng Xiao, Meng-Lan Zhou, Ying Yuan, Yao Wang, Li Zhang, Tian-Shu Sun, Ya-Ting Ning, Pei-Yao Jia, Wei Kang, Fanrong Kong, Sharon C.-A. Chen, Yanan Zhao, Ying-Chun Xu

**Affiliations:** ^1^Department of Clinical Laboratory, Peking Union Medical College Hospital, Chinese Academy of Medical Sciences, Beijing, China; ^2^Graduate School, Peking Union Medical College, Chinese Academy of Medical Sciences, Beijing, China; ^3^Beijing Key Laboratory for Mechanisms Research and Precision Diagnosis of Invasive Fungal Diseases (BZ0447), Beijing, China; ^4^Department of Central Laboratory, Peking Union Medical College Hospital, Chinese Academy of Medical Sciences, Beijing, China; ^5^Centre for Infectious Diseases and Microbiology Laboratory Services, ICPMR –New South Wales Health Pathology, The University of Sydney, Westmead, NSW, Australia; ^6^Center for Discovery and Innovation, Hackensack Meridian Health, Nutley, NJ, United States; ^7^Department of Medical Sciences, Hackensack Meridian School of Medicine, Nutley, NJ, United States

**Keywords:** *Aspergillus lentulus*, accurate identification, multi-drug resistance, fatal infections, China

## Abstract

Invasive aspergillosis (IA) due to *Aspergillus lentulus* is associated with high mortality. In this study, we investigated the clinical and microbiological characteristics of 6 fatal cases of proven or probable IA caused by *A. lentulus* in China. Underlying immunosuppression, prior antifungal exposure, and intensive care unit (ICU) hospitalization were important risk factors for invasive *A. lentulus* infection. Phenotypic differences were observed for *A. lentulus* isolates including slower growth, reduced sporulation, and inability to grow at 48°C, compared with *Aspergillus fumigatus complex*. ITS sequencing was unable to distinguish *A. lentulus* from *A. fumigatus*, but sequencing of the *benA*, *CaM*, and *rod A* loci enabled reliable distinction of these closely related species. Phylogenetic analysis further confirmed that the ITS region had little variation within the *Aspergillus* section Fumigati while the *benA* gene offered the highest intraspecific discrimination. Microsatellite typing results revealed that only loci on chromosomes 1, 3, 5, and 6b generated detectable amplicons for identification. All *A. lentulus* isolates showed *in vitro* resistance to multiple antifungal drugs including amphotericin B (MIC range 4 to 8 μg/ml), itraconazole (MIC 2 μg/ml), voriconazole (MIC of 4–16 μg/ml), and posaconazole (MIC of 0.5–1 μg/ml). However, MECs for the echinocandin drugs ranged from 0.03–0.25, ≤0.008–0.015, and ≤0.015–0.03 μg/ml for caspofungin, micafungin, and anidulafungin, respectively. *A. lentulus* is an emerging fungal pathogen in China, causing fatal disease, and clinicians as well as laboratories should be alert to their increasing presence.

## Introduction

Invasive aspergillosis (IA) remains a major invasive fungal infection with serious clinical consequences among immunocompromised patients (Sugui et al., 2014), of which the *Aspergillus fumigatus* complex is the most common cause. Notably, other causative *Aspergillus* spp. including *A. flavus*, *A. terreus*, and *A. niger* and cryptic species within the *A. fumigatus* complex have been increasingly recognized (Gurcan et al., 2013; Sugui et al., 2014). Of the last, in particular *Aspergillus lentulus* has become a significant pathogen in many countries including America, Spain, Argentina, Denmark, France, Germany, Turkey, Switzerland, Brazil, Japan, Australia, and Korea (Balajee et al., 2004, 2005a, 2009; Alcazar-Fuoli et al., 2008; Montenegro et al., 2009; Symoens et al., 2010; Mortensen et al., 2011; Zbinden et al., 2012; Datta et al., 2013; Escribano et al., 2013; Gurcan et al., 2013; Alastruey-Izquierdo et al., 2014; Lago et al., 2014; Bastos et al., 2015; Kidd et al., 2015; Tamiya et al., 2015; Yoshida et al., 2015; Tetsuka et al., 2017; Won et al., 2018).

*Aspergillus lentulus* was first described in 2004 as an opportunistic pathogen responsible for fatal infections in four hematopoietic stem cell transplant patients (Balajee et al., 2004; Balajee et al., 2005b). It is a sibling species of *A. fumigatus* within the *A. fumigatus* complex but with poor sporulating capacity, leading to diagnostic difficulty (Sugui et al., 2014; Lamoth, 2016). It tends to cause infection associated with higher mortality (over 60%) and poorer clinical outcome compared with that of *A. fumigatus* (Sugui et al., 2014; Won et al., 2018).

As such, in the clinical laboratory, *A. lentulus* often is misidentified as another *Aspergillus* species or simply identified only to genus level, by conventional morphological analysis (Symoens et al., 2010). Molecular methods are required for definitive species identification. Analysis of partial DNA sequences of various genes, such as the internal transcribed spacer (ITS) rDNA region, β-tubulin (*benA*), calmodulin (*CaM*), and rodlet A (*rodA*), have been reported as markers to differentiate different species within the *Aspergillus* section Fumigati (Lamoth, 2016). In addition, microsatellites [or short tandem repeats (STR)] have been considered as useful genetic tools in outbreak investigation and to delineate transmission routes of the *A. fumigatus* species complex with reproducibility and high discrimination power (Araujo et al., 2009, 2012). Compared to multi-locus sequence typing (MLST), microsatellite-based typing seemed to be more reproducible and cost-effective for *A. fumigatus* identification (Klaassen, 2009; Vanhee et al., 2009). Unfortunately, there are very few data on these methods in the characterization and study of genetic diversity in *A. lentulus* (Araujo et al., 2012).

The present study is the first to examine the clinical, microbiological, and molecular characteristics of *A. lentulus* and its infections from China.

## Materials and Methods

### Ethics Statement

The study was approved by the Human Research Ethics Committee of Peking Union Medical College Hospital (No. S-263). Written informed consent was obtained from all patients in the study and for permission to study the isolates cultured from them for scientific research.

### Patients and *A. lentulus* Isolates

A total of six non-duplicate *Aspergillus* isolates were recovered from the respiratory tract of six patients diagnosed with proven or probable IA (De Pauw et al., 2008; Cornely et al., 2019) under the China Hospital Invasive Fungal Surveillance Net (CHIF-NET; Wang et al., 2012)—Northern China program from January 2016 to December 2017. This program is a laboratory-based, multicenter study of invasive fungal diseases (De Pauw et al., 2008) including those caused by yeasts and filamentous fungi. A total of 80 hospitals in 6 provinces in the north part of China participated. The 580 isolates of filamentous fungi were collecting in the period, and the six *Aspergillus* isolates were confirmed as *A. lentulus* by rRNA sequencing at the central laboratory. Patients’ charts were reviewed to determine patient demographics, clinical features of infection including underlying disease/risk condition, prior antifungal therapy, treatment, and outcomes. All isolates were subcultured onto potato dextrose agar (PDA) and incubated at 28°C for 7–30 days prior to study. Isolates were identified to species complex level based on morphological characteristics (Larone, 2005) as well as growth at 48°C.

### Confirmation of Species

#### Matrix-Assisted Laser Desorption Ionization–Time of Flight Mass Spectrometry System

The identification of all the isolates was also undertaken using two Matrix-assisted laser desorption ionization–time of flight mass spectrometry (MALDI-TOF MS) systems—the bioMérieux Vitek MS (bioMérieux) and Bruker Autoflex Speed (Bruker Daltonics, Bremen, Germany). Preparation of *A. lentulus* isolates for MALDI-TOF MS identification was performed according to the manufacturer’s instructions with some modifications (Li et al., 2017). The acquisition and analysis of mass spectra were handled using software Myla (for Vitek MS, database version 3.2.0, bioMérieux) and Biotyper version 3.1 with the Filamentous Fungi Library 1.0 (for Autoflex Speed, Bruker Daltonics), again following the manufacturer’s instructions. All identification results displaying a single result with a confidence score ≥ 1.700 or a confidence value of 99.9% were considered acceptable for Bruker Biotyper MS and Vitek MS, respectively (Wang et al., 2016; Zhou et al., 2016).

#### Sequence-Based Identification and Tandem-Repeat Locus Identification

Genomic DNA extraction was performed with QIAamp DNA Mini Kit (QIAGEN 51306; Qiagen, Hilden, Germany) in accordance with the manufacturer’s instructions. For all isolates, DNA amplification of the ITS, *benA*, *CaM*, and *rodA* sequences was performed as previously described (Li et al., 2017). In order to evaluate genetic polymorphisms among *A. lentulus* isolates, eight primer pairs targeting eight microsatellite loci located on chromosomes 1, 2, 3, 5, 6a, 6b, 7, and 8 were selected (Araujo et al., 2009). The PCR products were sequenced in both directions using the ABI 3730XL system (Applied Biosystems, Foster City, CA, United States). Obtained ITS, *benA*, *CaM*, and *rodA* sequences were queried against those contained in the GenBank database under accession numbers NR135407, EF669825, EF669895, and HQ127311, using the nucleotide Basic Local Alignment Search Tool (BLASTn, http://blast.ncbi.nlm. nih.gov).

#### Phylogenetic Analysis

Nucleotide sequences of species closely related to *A. lentulus* in the *Aspergillus* section Fumigati including *A. fumigatus sensu stricto*, *A. udagawae*, *A. viridinutans*, *A. thermomutatus* (*Neosartorya pseudofischeri*), *A. novofumigatus*, and *A. hiratsukae* available in GenBank as of 30th August 2019 were downloaded (Lamoth, 2016). Phylogenetic analysis was performed with software MEGA (Molecular Evolutionary Genetic Analysis software, version 6.0; http://www.megasoftware.net) using the Neighbor-Joining (NJ) method, with all positions containing gaps and missing data eliminated from the data set. The significance of the cluster nodes was determined by bootstrapping with 1,000 randomizations *A. clavatus* sequences (GenBank accession NR121482, AF057312, HE661596, and AB524404) were used as outgroups.

#### Antifungal Susceptibility Testing

The *in vitro* susceptibility to nine antifungal drugs [fluconazole (FLZ), voriconazole (VOR), itraconazole (ITR), posaconazole (POS), caspofungin (CAS), micafungin (MCF), anidulafungin (AND), amphotericin B (AMB), and 5-flucytosine (5-FC)] was determined by broth microdilution methodology according to the Clinical and Laboratory Standards Institute (CLSI) M38-A3 protocol (Clinical and Laboratory Standards Institute [CLSI], 2017) and by Sensititre YeastOne^TM^ YO10 (SYO) methodology (Thermo Scientific, United States) following the manufacturer’s instructions. The MICs of AMB and azoles were read as the lowest concentration that resulted in no discernible growth following 48 h of incubation (Clinical and Laboratory Standards Institute [CLSI], 2017). The minimum effective concentrations (MECs) of CAS, AND, and MCF were read in accordance with the CLSI M38-A3 protocol (Clinical and Laboratory Standards Institute [CLSI], 2017). The quality control organisms were *Candida parapsilosis* ATCC22019 and *Candida krusei* ATCC6258. Due to the lack of clinical breakpoints or epidemiologic cutoff values (ECVs) for *A. lentulus*, current CLSI ECVs established for at the *A. fumigatus* species complex were used to classify the isolates as wild type (WT) or non–wild type (non-WT; Clinical and Laboratory Standards Institute [CLSI], 2018; Won et al., 2018).

#### Nucleotide Sequence Accession Numbers

The ITS region, *benA*, *CaM*, and *rodA* gene sequences of strain 16R08468 (isolated from patient 6), 16H1047 (isolated from patient 2), 16H1006 (isolated from patient 1), 17R16664 (isolated from patient 4), 17Z31741 (isolated from patient 5), and 17R16195 (isolated from patient 3) have been deposited in GenBank with accession numbers MN235864 to MN235869, MN275499 to MN275504, MN275505 to MN275510, and MN275511 to MN275516, respectively, (see Table 1).

**TABLE 1:** Main clinical aspects of invasive aspergillosis caused by *Aspergillus lentulus* in this study.

Patient	Strain ID	City/Hospital	Age, y/Sex	Depart-ment	Certainty of diagnosis	Underlying disease/risk condition	Specimen type/collec-tion site	Specimen collection date	Initial identifi-cation	BDG test results, date	GM test results/date	Prior antifungal therapy	Immuno-suppressive drugs	Outcome
1	16H1006	Harbin/H1	47/F	Rheuma-tology	Proven IA	RA	Sputum/lung	2016/1/13	*Aspergillus fumigatus*	646, 2016/1/22	0.67, 2016/1/22	Unknown	Yes	Unknown
2	16H1047	Harbin/H1	72/F	Thoracic surgery	Proven IA	RA	Sputum/lung	2016/5/25	*Aspergillus fumigatus*	673.2, 2016/6/1	0.66, 2016/6/1	Unknown	Yes	Unknown
3	17R16195	Beijing/PU	61/M	ICU	PCP, CMV pneumonia, probable IA	ANCA associated systematic Vasculitis	Sputum/lung	2017/10/11	*Aspergillus fumigatus complex*	173.4, 2017/10/11	No	Caspofungin	Yes	Death
4	17R16664	Beijing/PU	25/F	ICU	Proven IA	Takayasu’s arteritis, MOF, shock	Sputum/lung	2017/10/18	*Aspergillus fumigatus complex*	193.2, 2017/10/19	No	Amphotericin B, voriconazole	Yes	No cure, discharged against medical advice
5	17Z31741	Beijing/PU	39/F	ICU	Pulmonary infection, probable IA	Respiratory failure, intracranial infection	Tracheal intubation aspirate/lung	2017/10/11	*Aspergillus fumigatus complex*	No	No	No	Yes	death
6	16R08468	Beijing/PU	29/F	ICU	Proven IA	Septic shock, severe pneumonia, MOF, DIC	BALF/lung	2016/5/21	*Aspergillus sp.*	185.2, 2016/5/23	No	Caspofungin	Yes	death

*ICU, intensive care unit; IA, invasive aspergillosis; RA, rheumatoid arthritis; ANCA, anti-neutrophil cytoplasmic antibodies; MOF, multiple-organ failure; DIC, disseminated or diffuse intravascular coagulation; and BALF: bronchoalveolar lavage fluid.*

## Results

### Clinical Characteristics

All six patients with proven or probable *A. lentulus* infection were immunocompromised or had significant comorbidities such as rheumatoid arthritis and anti-neutrophil cytoplasmic antibodies (ANCA)-associated systemic vasculitis and/or were suffering from multiple-organ failure (MOF), shock, and disseminated intravascular coagulation (DIC; Table 1). Five patients were females, and four were in ICU when infection developed. All patients had received immunosuppressive therapy comprising corticosteroids and/or cyclophosphamide. There was no relevant data on prior antifungal exposure or clinical outcome information for patients 1 and 2; the other four were considered to have poor prognosis (Table 1). Three patients received prior antifungal therapy including caspofungin in two patients and amphotericin B combined with voriconazole in the other. Because the first sign, symptom, or finding of invasive fungal infection was occurring before antifungal drug treatment, they should not be classified as breakthrough IFI (Cornely et al., 2019). Five patients had obviously increased (1,3)-β-d-glucan (BDG test, Dynamiker, China), all with O.D > readings of over 100 pg/ml. Galactomannan (GM) was either unknown or not tested in these patients.

### Phenotypic Characterization of the *A. lentulus* Isolates

**FIGURE 1:**
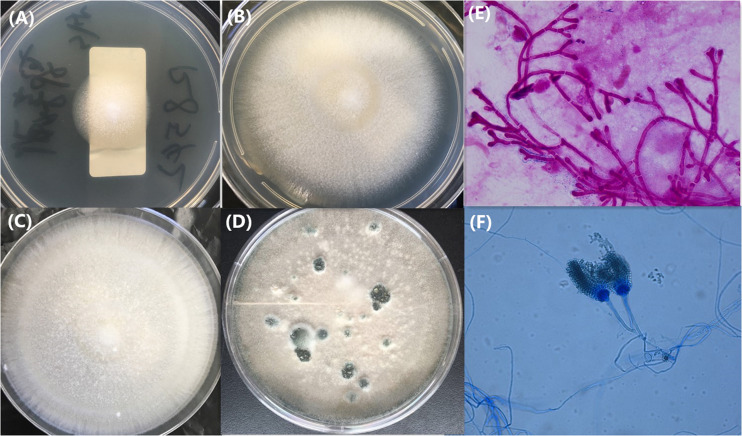
Colony appearance of *A. lentulus* isolates on Potato Dextrose Agar **(A–D)** and Gram’s staining of the bronchoalveolar lavage fluid specimen **(E)** and microscopic appearance of the branched conidiophores on Potato Dextrose Agar by lactophenol cotton blue staining **(F)**. Incubation conditions: 1A, 28°C, 2 days; 1B, 28°C, 5 days; 1C, 28°C, 7 days; 1D, 28 days; and 1F, 28°C, 7 days. The isolate of **(A–F)** selected the isolates of 16R08468.

All six isolates were slow growers and poor sporulators on PDA at 28 and 35°C and failed to grow at 48°C. After 7 days of incubation on PDA, all isolates grew as colored white and velvety colonies mainly consisting of hyphae interspersed with sporadic gray-green spores (Figure 1). After prolonged incubation (8 to 21 days) on PDA, the colonies began sporulating, which had the same appearance as typical colonies of *A. fumigatus* (Figure 1). Direct microscopic analysis of sputum or bronchoalveolar lavage fluid revealed abundant septate fungal hyphae septum with acute angle branching, arranged radially or coral like (Figure 1E). Microscopic examination on day 3 to day 21 showed stipes, head, and conidia of *A. lentulus*. Uniseriate conidial heads are columnar, and conidia are produced in basipetal succession forming long chains and are globose to broadly ellipsoidal. Conidiophore stipes are smooth-walled and have subglobose vesicles. Because of near identical features to *A. fumigatus* sensu stricto, the two may be confused and misidentified by microscopic examination (James et al., 2011; Figure 1F).

### Sequence-Based Identification

All isolates had identical ITS and *CaM* sequences, but there were three single-nucleotide variations including C183T, C268T, and C347T of the *benA* sequence for two of the isolates. In addition, isolate 16H1047 had a unique *rod A* sequence with 7-base nucleotide changes including C to G in position 257; T to C in position 260, 283, 347, and 403; G to T in position 383; and C to T in position 365 compared with other isolates. All isolates were identified by ITS sequencing as “*A. lentulus*” or “*A. fumigatus*” with 100% sequence identity. Of note, *Ben A* and *CaM* gene sequencing analysis successfully identified all isolates as *A. lentulus* with 100% sequence identity. Except for isolate 16H1047 which was identified as *A. lentulus* or as *A. fumigatus* with 100% sequence identity, the other five isolates were successfully identified as *A. lentulus* by *rod A* sequencing.

### Tandem-Repeat Locus Identification

Among eight microsatellite loci used, only four (located on chromosomes 1, 3, 5, and 6b) were detected by standard electrophoresis. PCR amplification of the other four microsatellite loci located in chromosome 2, 6a, 7, and 8 did not generate any detectable amplicon. All six *A. lentulus* isolates had identical peaks at positions 830, 206, 240, and 208 bp corresponding to each microsatellite locus of chromosomes 1, 3, 5, and 6b. This result clearly differs from that of *A. fumigatus*, which shows an expected electrophoretic profile of 8 peaks, corresponding to each microsatellite locus (Araujo et al., 2012). Furthermore, the results may indicate that the set of eight microsatellite loci had high ability to distinguish *A. fumigatus* and *A. lentulus* but low discrimination power or poor polymorphism to genotype in the *A. lentulus* species.

### MALDI-TOF MS Analysis and Antifungal Susceptibilities

Mass spectrometry spectra of *A. lentulus* were not contained in the Bruker Biotyper database but were in the Vitek MS database. Hence, the Bruker Biotyper system provided “no identification” (log score < 1.70) for all *A. lentulus* isolates, while the Vitek MS system correctly identified all the isolates to species level with a confidence value of 99.9 (Table 2).

**TABLE 2:** Results of MALDI-TOF MS identification and antifungal susceptibility for *Aspergillus lentulus* isolates.

Patient	Strain ID	Identification by the MALDI- TOF MS system (score)^a^		Susceptibility profile (μ g/ml)																	
		Bruker biotyper^b^	Vitek MS^b^	CAS		MCF		AND		5-FC		POS		VOR		ITR		FLZ		AMB	
				CL	SYO	CL	SYO	CL	SYO	CL	SYO	CL	SYO	CL	SYO	CL	SYO	CL	SYO	CL	SYO
1	16H1006	No ID^c^	*A. lentulus*(99.9)	0.12	0.06	0.015	≤0.008	0.03	0.03	>64	>64	0.5	0.5	8	4	2	2	>256	>256	4	8
2	16H1047	No ID	*A. lentulus*(99.9)	0.12	0.06	≤0.008	≤0.008	≤0.015	≤0.015	>64	>64	1	0.5	8	4	2	2	>256	>256	8	8
3	17R16195	No ID	*A. lentulus*(99.9)	0.25	0.12	0.015	≤0.008	0.03	≤0.015	>64	>64	0.5	1	8	8	2	2	>256	>256	4	8
4	17R16664	No ID	*A. lentulus*(99.9)	0.03	0.03	0.015	≤0.008	0.03	≤0.015	>64	>64	1	0.5	16	8	2	2	>256	>256	4	8
5	17Z31741	No ID	*A. lentulus*(99.9)	0.12	0.06	≤0.008	≤0.008	0.03	≤0.015	>64	>64	1	0.5	8	4	2	2	>256	>256	4	8
6	16R08468	No ID	A. lentulus(99.9)	0.12	0.06	≤0.008	≤0.008	≤0.015	≤0.015	>64	>64	0.5	0.5	8	4	2	2	>256	>256	4	8

*^a^MALDI-TOF MS: matrix-assisted laser desorption ionization–time of flight mass spectrometry. ^b^The analysis of mass spectra was performed by Bruker Biotyper version 3.1 with the Filamentous Fungi Library 1.0 (Bruker Daltonics) and software Myla for Vitek MS (database version 3.2.0, bioMérieux). ^c^No ID: no identification. CL: the Clinical and Laboratory Standards Institute (CLSI); SYO: Sensititre YeastOne^TM^ YO10.*

Table 2 also shows the MIC or MEC values for the six isolates. The MEC range for CAS, MCF, and AND were 0.03 to 0.25 or 0.03 to 0.12, ≤0.008 to 0.015 or ≤0.008, and ≤0.0015 to 0.03 μg/ml by CLSI or SYO, respectively. According to CLSI ECVs (Clinical and Laboratory Standards Institute [CLSI], 2018), all were classified as wild type (WT) for the echinocandins. In contrast, all the isolates showed *in vitro* resistance to multiple drugs in other antifungal classes based on both methods, including AMB (MIC range 4 to 8 μg/ml), ITR (MIC 2 μg/ml), VOR (MIC range 4 to 16 μg/ml), POS (MIC range 0.5 to 1 μg/ml), and FLZ (MIC > 256 μg/ml), according to CLSI ECVs, for *A. fumigatus*.

### Phylogenetic Analysis

**FIGURE 2:**
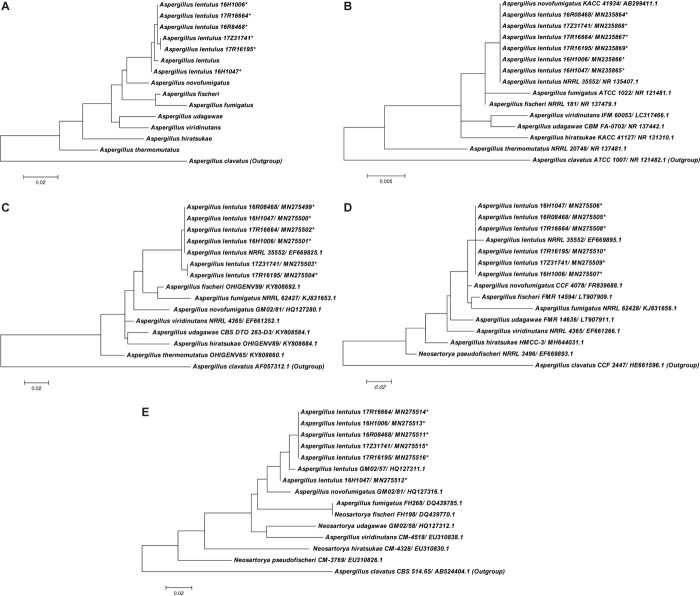
The Neighbor-Joining (NJ) tree of *A. lentulus* generated from *A. lentulus* for collaboration with ITS, *ben A*, *CaM*, and *rod A* sequences **(A)** and individual gene of ITS **(B)**, *ben A* **(C)**, *CaM* **(D)**, and *rod A* **(E)** from this study and other six closely related isolates in the *Aspergillus* section Fumigati which are most frequently recovered in clinical specimens and associated with invasive fungal diseases available in GenBank. *A. clavatus* (GenBank accession NR121482, AF057312, HE661596, and AB524404) were used as outgroups. *The isolates were collected from this study.

A total of nine closely related species in the *Aspergillus* section Fumigati were employed to construct phylogenetic trees (Figure 2). Phylogenetic analysis based on gene sequences from the combination of the ITS region and *benA*, *CaM*, and *rodA* loci together or each single gene alone showed that the six *A. lentulus* isolates in our study form a monophyletic cluster with the *A. lentulus*-type strain, confirming their original identification. Focusing on the phylogenetic tree constructed from all four loci, *A. lentulus* isolates were not only clearly distinguished from other species in the *Aspergillus* section Fumigati but had intraspecies polymorphisms. It was observed that 17Z31741 and 17R16195 were more closely related and 16H1047 was the most isolated, compared to other isolates in the *A. lentulus* species in the four loci phylogenetic tree. This was due to diverse *ben A* sequences of 17Z31741 and 17R16195 and a unique *rod A* sequence of 16H1047. Single-gene phylogenetic analysis revealed that ITS sequencing was unable to distinguish *A. lentulus* from *A. novofumigatus*, but *benA*, *CaM*, and *rodA* sequences were all able to separate *A. lentulus* from the above species in a single monophyletic cluster. Of the three loci, *benA* had the highest intraspecific discrimination in the nine closely related species in the *Aspergillus* section Fumigati.

## Discussion

*Aspergillus lentulus* is described as being inherently resistant to azole drugs, and infections due to this cryptic species are rising globally. Most isolates of this species have been recovered from hematopoietic stem cell transplant, heart transplant, and kidney transplant recipients (Bastos et al., 2015). Here we present for the first time six cases of *A. lentulus* infection from China.

In our study, all the patients were diagnosed as proven or probable IA (De Pauw et al., 2008). All suffered from autoimmune disease and/or were severely ill. The majority of patients were ICU patients with prior antifungal therapy and had fatal outcome. We found that immunocompromised condition, prior antifungal therapy, and ICU hospitalization were important risk factors for invasive *A. lentulus* infection, consistent with previous findings (Yagi et al., 2019).

Phenotypically, *A. lentulus* isolates exhibited differences in growth characteristics compared to *A. fumigatus*, including slower growth, reduced sporulation, and inability of growth at 48°C (Montenegro et al., 2009; Zbinden et al., 2012). Recent improvement in MALDI-TOF MS-based databases has provided a promising alternative for routine identification of *A. lentulus* and other *A. fumigatus*-related species within a limited time frame (Verwer et al., 2014; Lamoth, 2016). In our study, the Vitek MS platform (bioMérieux) enabled identification of *A. lentulus*, whereas the Bruker Biotyper system did not, because currently the reference mass spectra of *A. lentulus* are not available. Further investigations for pretreatment procedures of filamentous fungi, spectra characterization, and the establishment of reference database are required (Lamoth, 2016).

We found that accurate identification to species level of *A. lentulus* can be achieved by sequencing of genetic markers. However, ITS sequencing was not sufficiently discriminatory to distinguish *A. lentulus* from *A. fumigatus sensu stricto*. In contrast, both *benA* and *CaM* were good markers for *A. lentulus* species identification. This finding also supports previous comparative sequence analyses of the ITS region for intersection identification of *Aspergillus* spp. and of the beta-tubulin or calmodulin genes for intrasection identification at the species level (Lamoth, 2016). Interestingly, the *rodA locus* alone was not reliable for *A. lentulus* identification. Isolate 16H1047 harbored a 7-SNP difference in *rodA* compared to other isolates in this study, resulting in an ambiguous identification of either “*A. lentulus”* or “*A. fumigatus*” using *rodA.* The higher variance of the *rod A* sequences of *A. lentulus* may result in a lower discriminatory resolution of this gene. Phylogenetic analysis of nine closely related species in the *Aspergillus* section Fumigati based on each single gene further confirmed that the ITS sequence was highly conserved among *Aspergillus* section Fumigati, while the *benA* gene had the highest intraspecific discrimination among the four genes.

The employment of microsatellite typing may be appropriate for resolving the origin of outbreak episodes and investigation of phylogenetic patterns (de Valk et al., 2007; Thierry et al., 2010). Thierry et al. (2010) have proposed a panel of eight microsatellites with high discriminatory power for genotyping *A. fumigatus*. Our study revealed that only loci in chromosomes 1, 3, 5, and 6b generated detectable amplicons for identification. Our results also demonstrated that microsatellite markers on chromosomes 1, 3, 5, and 6b seem to be discriminatory in differentiating *A. lentulus* from other species within *section* Fumigati, but it may lack intraspecies variation to distinguish different origins of *A. lentulus*. In order to determine the discrimination capacity of all eight microsatellite markers among *section* Fumigati, even within *A. lentulus* species, incorporating more species *Aspergillus section* Fumigati or a larger number of each species is necessary.

**FIGURE 3:**
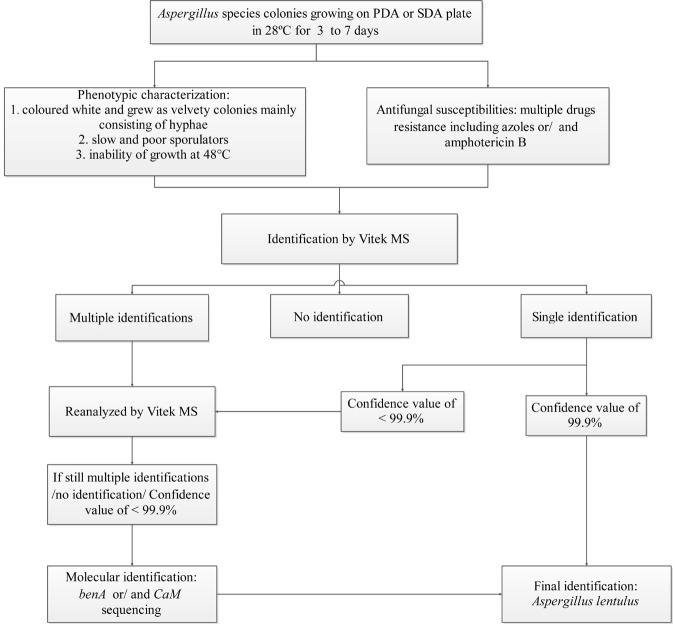
An identification algorithm for *A. lentulus* species based on the Vitek MS and selective molecular identification. Abbreviation: PDA, Potato Dextrose Agar; SDA, Sabouraud Dextrose Agar.

Based on our findings, we designed an identification algorithm for clinical laboratories to identify *A. lentulus* species with high accuracy (Figure 3). The trigger to adopt this algorithm is when colonies are suspected for *A. lentulus*, i.e., colonies are colored white, are of a velvety texture, mainly consist of hyphae, and grow slowly with poor sporulation. Further, inability to grow at 48°C and exhibiting multidrug resistance to azoles or/and amphotericin B increase the likelihood of *A. lentulus*. MALDI-TOF MS using the Vitek MS system (bioMérieux) and molecular identification are needed for definitive identification (see Figure 3 for conditions required for MALDI TOF MS identification). *Ben A* or/and *CaM* sequencing are recommended as the preferred gene loci for molecular diagnostics. It needs to be pointed out that the molecular method enables final identification for *A. lentulus*, regardless of MALDI TOF MS results.

Similar to *A. fumigatus*, *A. lentulus* showed high resistance to fluconazole, with MIC over 256 μg/ml. Moreover, *in vitro* susceptibility results showed that all *A. lentulus* isolates were resistant to amphotericin B and azole drugs yet remain susceptible to echinocandins. The MIC values of the azoles (mainly voriconazole and itraconazole) and amphotericin B in our study were higher than those in previous studies (Balajee et al., 2004, 2009; Montenegro et al., 2009; Symoens et al., 2010; Zbinden et al., 2012; Bastos et al., 2015; Tamiya et al., 2015; Won et al., 2018), while the MEC values of echinocandins were either similar to Kidd et al. (2015), Yoshida et al. (2015), and Won et al. (2018), or lower than those previously reported (Balajee et al., 2004; Alhambra et al., 2008; Montenegro et al., 2009; Symoens et al., 2010). The 2016 Infectious Diseases Society of America (IDSA) guidelines establish voriconazole as the primary antifungal choice for IA treatment; voriconazole combined with an echinocandin may be considered in documented IA patients (Patterson et al., 2016). However, *A. lentulus* has generally proven to possess evaluated MIC values to voriconazole and other azoles (Balajee et al., 2004, 2009; Montenegro et al., 2009; Symoens et al., 2010; Zbinden et al., 2012; Bastos et al., 2015; Tamiya et al., 2015; Won et al., 2018). More importantly, poor clinical outcome has been associated with azole therapy administered to patients with IPA caused by *A. lentulus* (Yoshida et al., 2015; Tetsuka et al., 2017). Tetsuka et al. (2017) chose micafungin to cure *A. lentulus* infection after no clinical response was observed with voriconazole use. Of note, even though all *A. lentulus* isolates in our study were susceptible to echinocandins *in vitro*, two patients died after receiving caspofungin treatment. The mechanisms of azole resistance in *A. lentulus* are Cyp51A dependent but are different from what has been described previously for *A. fumigatus* (Mellado et al., 2011). Molecular dynamics modeling revealed that some critical differences were observed in the putative closed form adopted by the proteins upon voriconazole binding in Cyp51A. Some major differences in the protein’s BC loop could differentially affect the lockup of voriconazole, which in turn could correlate with *A. lentulus* differences in azole susceptibility (Alcazar-Fuoli et al., 2011).

In summary, we evaluated the clinical, microbiological, and molecular features of invasive *A. lentulus* infections in Chinese patients. In particular, we highlight the importance of accurate identification with susceptibility testing of *Aspergillus* species for appropriate therapy. Molecular methods are needed for accurate speciation and further characterization of this fungal species.

## Data Availability Statement

The datasets generated for this study are available on request to the corresponding author.

## Ethics Statement

The study was approved by the Human Research Ethics Committee of Peking Union Medical College Hospital (No. S-263). Written informed consent was obtained from all patients in the study, and for permission to study the isolates cultured from them for scientific research. Written, informed consent was obtained from the individual(s) and next of kin for the publication of any potentially identifiable images or data included in this article.

## Author Contributions

S-YY, L-NG, MX, and Y-CX conceived and designed the experiments. S-YY, YY, YW, LZ, T-SS, Y-TN, P-YJ, and WK performed the experiments. S-YY, M-LZ, and YZ performed the data analysis and wrote the manuscript. FK, YZ, and SC revised the manuscript critically for important intellectual content. All authors participated in the critical review of this manuscript.

## Conflict of Interest

The authors declare that the research was conducted in the absence of any commercial or financial relationships that could be construed as a potential conflict of interest. The reviewer MH declared past co-authorship with one of the authors SC to the handling editor.
